# An association between liraglutide treatment and reduction in excessive daytime sleepiness in obese subjects with type 2 diabetes

**DOI:** 10.1186/s12902-015-0074-6

**Published:** 2015-12-04

**Authors:** Fernando Gomez-Peralta, Cristina Abreu, Jose Carlos Castro, Elvira Alcarria, Margarita Cruz-Bravo, Maria Jesús Garcia-Llorente, Cristina Albornos, Concepción Moreno, María Cepeda, Francisca Almodóvar

**Affiliations:** Unidad de Endocrinología y Nutrición, Hospital General de Segovia, C/ Miguel Servet s/n, 40002 Segovia, Spain; Servicio de Medicina Interna, Hospital General de Segovia, Segovia, Spain; Unidad de Endocrinología y Nutrición, Hospital de Alcorcón, Madrid, Spain

**Keywords:** Excessive daytime sleepiness, Abnormal sleep patterns, Obstructive sleep apnoea, Type 2 diabetes, Obesity, Liraglutide, Glucagon-like peptide-1

## Abstract

**Background:**

The main purpose of the present study is to evaluate whether treatment with long-acting human glucagon-like peptide-1 liraglutide was associated with an improvement of excessive daytime sleepiness (EDS) in obese subjects with type-2 diabetes.

**Methods:**

This single-centre retrospective study included 158 obese (body mass index [BMI] ≥ 30 kg/m^2^) adult subjects with type-2 diabetes who were initiated with liraglutide treatment at least 3 months before study inclusion. Data of the Epworth Sleepiness Scale (ESS), anthropometric parameters, glucose-control and metabolic parameters were collected at liraglutide initiation (baseline) and at months 1 and 3 after liraglutide initiation.

**Results:**

Significant reductions in ESS score were achieved at months 1 (−1.3 ± 2.8, *p* < 0.001) and 3 (−1.5 ± 3.0, *p* < 0.001) after liraglutide introduction. After 3 months of treatment with liraglutide, significant changes in body weight (*p* < 0.001), BMI (*p* < 0.001), waist (*p* < 0.001) and neck circumferences (*p* < 0.005), HbA1c (*p* < 0.001), mean blood glucose (*p* < 0.001), fasting plasma glucose (*p* < 0.001), triglycerides (*p* < 0.01) and total cholesterol (*p* < 0.001) were achieved.

**Conclusions:**

After 3 months of treatment with liraglutide a significant reduction in EDS was observed in obese subjects with type-2 diabetes. Besides this, significant changes in body weight and metabolic parameters of diabetes control were also accomplished. Further investigation is required to determine whether liraglutide could improve other abnormal sleep patterns and obstructive sleep apnoea.

## Background

Abnormal sleep patterns (ASPs), characterized by short or long durations of sleep and excessive daytime sleepiness (EDS), have a considerable impact on an individual’s health [1]. Obstructive sleep apnea (OSA) is the most common form of ASP, characterized by the repetitive complete or partial collapse of the upper airway during sleep [2]. There is a growing body of literature describing the association between ASPs and the subsequent development of type-2 diabetes, even in non-obese individuals [3–7]. EDS is more prevalent in subjects with diabetes and in subjects with insulin resistance, a well-known *prediabetic* condition [8, 9]. Additionally, OSA has also been shown to impact on glycemic control among diabetic patients, independently of obesity [10]. Recently, a study concluded that impaired sleep quality and daytime sleepiness are associated with decreased diabetes self-management in adults with type 2 diabetes [11]. EDS has been also related to severe hypoglycemia in subjects with type 2 diabetes in the Edinburgh type 2 diabetes study [12]. Quality of life and life expectancy could be affected by this condition in this population.

In a number of clinical studies, bariatric surgery-induced weight loss in obese subjects was associated with a dramatic improvement in subjective sleepiness, as well as other associated comorbidities [13–15]. EDS was also reduced with a weight reduction program, including dietary counselling and behavioral change support [16].

Liraglutide, a long-acting human glucagon-like peptide-1 receptor agonist (GLP-1ra) [17], is indicated for the treatment of type 2 diabetes. Liraglutide exerts its potent glucose-lowering effect through different mechanisms that lead to clinically significant reductions in glycated haemoglobin (HbA1c) and body weight [18]. A reduction of insulin resistance has been proved in patients with type 2 diabetes treated with liraglutide [19].

Lower body weight, decreased insulin resistance and an improvement in glycaemic control could ameliorate the perceived quality of life of subjects with type 2 diabetes and, to some extent, to relieve EDS.

Therefore, all the above prompted us to evaluate whether treatment with GLP-1ra liraglutide was associated with an improvement of EDS in obese subjects with type-2 diabetes in routine clinical practice setting.

## Methods

This retrospective observational study was carried out at the Hospital General de Segovia (Segovia, Spain) in accordance with the Declaration of Helsinki, including all amendments. It was approved on February 4^th^ 2012 by the Clinical Research and Ethics Commission of the Hospital General de Segovia (Study code SEE-DIA-2012-11). All participants provided written informed consent to use their data prior to the study.

### Study subjects

The study population comprised obese (BMI ≥ 30 kg/m^2^) adult subjects with a documented diagnosis of type 2 diabetes who were initiated on liraglutide (Victoza®, Novo Nordisk, Bagsvaerd, Denmark) treatment at least 3 months before study inclusion. A pre-existing clinical diagnosis of OSA was not deemed necessary for inclusion. No patient was treated with continuous positive airway pressure (CPAP) during this study period. Subjects were excluded if they had undergone bariatric surgery or presented weight loss > 5 % within the previous 6 months. Patients with cognitive impairment were also excluded.

### Measurements

Following routine clinical practice, anthropometric parameters, glycaemic control and metabolic measurements, and the Epworth Sleepiness Scale (ESS) score data were retrospectively obtained from medical records at three time points: at liraglutide initiation (baseline) and at months 1 and 3 after liraglutide initiation.

#### Anthropometric parameters

Height (m) and weight (kg) were recorded. Body mass index (BMI) was calculated as weight/height squared (kg/m^2^). Waist circumference was measured at umbilical level in standing positions with a non-stretchable tape (cm). Also, body composition measurements were determined by bioelectrical impedance.

#### Glucose control measurements and metabolic parameters

Glucose control measurements included HbA1c, mean blood glucose (MBG) from self-monitored blood glucose profiles and fasting plasma glucose (FPG). The metabolic parameters included the collection of high-density-lipoprotein cholesterol (HDL), low-density-lipoprotein cholesterol (LDL), total cholesterol (TC) and triglycerides (TG) levels.

#### Epworth Sleepiness Scale

The ESS score was used to measure the clinical impact of EDS. It is defined as a simple and self-administered questionnaire which provides a measurement of the subject’s general level of sleep propensity and likelihood of dozing off in eight different real-life situations. All items are rated on a 4-point Likert scale, 0 = would never doze, 1 = slight chance of dozing, 2 = moderate chance of dozing, 3 = high chance of dozing. Scores vary from 0 to 24, with higher scores indicating higher levels of sleepiness [20]. At present, there are no universally adapted cut-off ranges for the ESS, but Drager et al. determined the sensitivity and specificity of ESS in predicting OSA (defined as ESS score >10) as 49 % and 80 %, respectively [21]. According to domain experts, we considered a 1-point reduction in ESS score to be clinically significant [22].

### Statistical considerations

The values are expressed as mean ± standard deviation (SD) for quantitative variables and as percentages for categorical variables. Means were compared with *T-test* and the correlation analyses were assessed with the Pearson/Spearman correlation coefficient. The significance level was determined at *p* < 0.05 and all statistical analyses were carried out with the statistical package SPSS v.18.0 (SPSS, Inc., Chicago, IL).

## Results

### Subjects characteristics

Between March 2012 and June 2013, a total of 163 subjects were included. Four subjects were excluded due to screening failure (*n* = 2) and incomplete data (*n* = 2), and another subject did not meet all the selection criteria. Thus, the evaluable population comprised 158 subjects, whose clinical and metabolic parameters are listed in Table 1. At baseline, mean ESS score was 5.9 ± 4.5 and 26 (17.4 %) subjects reached an ESS > 10.

**Table 1:** Baseline characteristics (*N* = 158)

Characteristics	Value
Age (years), mean ± SD	58.2 ± 11.5
Gender, n (%)	
Male	78 (49.4)
Female	80 (50.6)
Duration of type 2 diabetes (years), mean ± SD	11.0 ± 7.2
BMI (kg/m^2^), mean ± SD	38.1 ± 6.6
Waist perimeter (cm), mean ± SD	119.2 ± 13.7
Body fat (%), mean ± SD	40.6 ± 7.7
HbA1c (mmol/mol), mean ± SD	68 ± 16
MBG (mmol/L), mean ± SD	10.8 ± 2.4
FPG (mmol/L), mean ± SD	10.1 ± 3.6
HDL (mmol/L), mean ± SD	1.1 ± 0.3
LDL (mmol/L), mean ± SD	2.7 ± 1.0
TC (mmol/L), mean ± SD	4.8 ± 1.3

*BMI* body mass index, *FPG* fasting plasma glucose, *HbA1c* haemoglobin A1c, *HDL* high-density-lipoprotein cholesterol, *LDL* low-density-lipoprotein cholesterol, *MBG* mean blood glucose, *SD*  standard deviation,  *TC* total cholesterol

Subjects were initiated with liraglutide treatment injected subcutaneously from the starting dose of 0.6 mg/day and were increased over one week to 1.2 mg/day by the attending physicians.

### Changes of ESS score, anthropometric data, glucose-control measurements and metabolic parameters

**Fig. 1:**
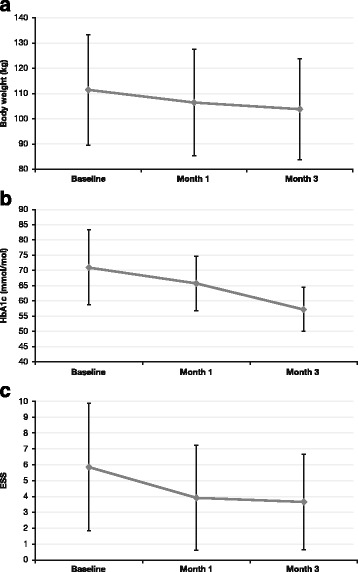
Evolution of body weight (**a**), HbA1c (**b**) and ESS score (**c**)

The evolution of body weight, HbA1c and ESS score from baseline to months 1 and 3 are depicted in Fig. 1. Pairwise comparisons showed that the ESS score significantly decreased from baseline to month 1 (6.3 ± 4.6 *vs* 4.9 ± 3.9; *p* < 0.001) and from baseline to month 3 (5.7 ± 4.4 *vs* 4.2 ± 3.6; *p* < 0.001). After three months of treatment with liraglutide, significant reductions were achieved in the means of body weight (102.8 ± 17.9 kg *vs* 98.4 ± 16.9 kg, *p* < 0.001), BMI (39.6 ± 6.8 kg/m^2^*vs* 37.9 ± 6.4 kg/m^2^; *p* < 0.001), waist circumference (122.1 ± 14.0 cm *vs* 118.9 ± 13.3 cm; *p* < 0.001) and neck circumference (42.2 ± 3.3 cm *vs* 40.8 ± 3.3 cm; *p* < 0.005), while the body fat percentage hardly changed (42.1 ± 7.6 % *vs* 41.2 ± 8.1 %; *p* = 0.173). Comparison of glucose-control measurements and metabolic parameters between baseline and month 3 are presented in Table 2.

**Table 2:** Pairwise comparison of glucose-control measurements and metabolic parameters after 3 months of treatment with liraglutide

	Baseline (mean ± SD)	Month 3 (mean ± SD)	*p*-value
HbA1c (mmol/mol)	68 ± 16	56 ± 14	<0.001
MBG (mmol/L)	10.8 ± 2.4	9.1 ± 2.0	<0.001
FPG (mmol/L)	10.7 ± 4.1	7.9 ± 2.1	<0.001
TG (mmol/L)	2.7 ± 3.7	1.7 ± 1.2	<0.01
HDL (mmol/L)	1.0 ± 0.2	0.9 ± 0.2	NS
LDL (mmol/L)	3.0 ± 1.1	2.6 ± 0.6	NS
TC (mmol/L)	5.1 ± 1.2	4.3 ± 0.7	<0.001

*FPG* fasting plasma glucose, *HbA1c* haemoglobin A1c, *HDL* high-density-lipoprotein cholesterol, *LDL* low-density-lipoprotein cholesterol, *MBG* mean blood glucose, *NS* not significant, *SD*  standard deviation, *TC* total cholesterol, *TG* triglycerides

**Fig. 2:**
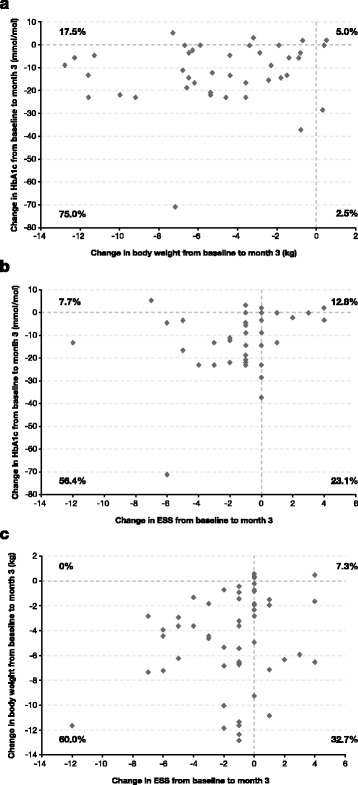
Scatter plots of changes in HbA1c and body weight (**a**), HbA1c and ESS (**b**) and body weight and ESS (**c**) from baseline to month 3 of treatment with liraglutide

The relative changes in HbA1c and body weight, HbA1c and ESS, as well as in body weight and ESS from baseline to month 3 after liraglutide initiation are presented as scatter plots in Fig. 2a, b and c, respectively. Overall, the majority of the study subjects were located in the lower left quadrant, i.e. HbA1c, body weight, and ESS score decreased for these individuals.

### Correlation of ESS score with the analytical parameters and the anthropometric measures

Baseline ESS score was negatively correlated with baseline body weight, BMI, waist perimeter, percentage of body fat, and HDL levels (Table 3). However, the only variable associated with changes in ESS score was ‘reductions in body weight’ (*r* = 0.269, *p* < 0.05). The rest of the parameters did not achieve statistical significance.

**Table 3:** Correlations between baseline parameters and ESS score

	Baseline ESS score	
	*r*	*p*-value
Body weight	−0.179	<0.05
BMI	−0.179	<0.05
Waist circumference	−0.175	<0.05
Body fat	−0.188	<0.05
HDL	−0.224	<0.05

*BMI* body mass index, *ESS* Epworth Sleepiness Scale, *HDL* high-density-lipoprotein cholesterol

## Discussion

To the best of our knowledge, this is the first study showing an association of liraglutide-induced body weight reduction and an improvement of EDS in obese subjects with type 2 diabetes in routine clinical practice. Recent scientific contributions are suggesting that ASPs, which includes EDS, are not only related to impaired health-related quality of life, but also influence the microvascular complications of diabetes (i.e. diabetic neuropathy) [23].

Within three months of therapy with liraglutide, significant reductions were achieved not only in body weight and BMI, but also in waist and neck circumferences. Concurrently, ESS score was reduced following one month of follow-up and was maintained after three months.

In our study, baseline anthropometric measures of obesity were negatively correlated with baseline ESS score. Some previous studies showed that the association between diabetes or insulin resistance and EDS was independent of obesity [8, 9]. As an example, Dixon et al. found that anthropometric measures explained only 3 % of ESS score variance in 1055 subjects presenting for obesity surgery [8]. Although our study population comprised a sample of very obese (mean BMI, 38.1 kg/m^2^) subjects with type 2 diabetes, due to the reimbursement policy for liraglutide prescription in Spain, it is possible that obesity in this particular group of patients was not determinant for baseline EDS. Despite changes in ESS score were shown to correlate positively with the reduction in body weight, no significant relation was achieved with the change in total body fat mass. This finding is consistent with earlier studies showing similar reductions in ESS score with surgical or non-pharmacological interventions reducing body weight [13–16].

Additionally to the direct effect of weight loss in EDS, a positive impact on perceived wellbeing or mood could be implicated in this improvement, because EDS was associated with poor energy and symptoms of depression in previous studies [7].

Liu et al. showed that EDS diagnosed with ESS, was prevalent amongst the insulin-resistant subgroup of obese individuals in comparison to their weight-matched insulin-sensitive counterpart [24]. We measured two surrogate markers of insulin resistance: HDL-cholesterol levels and waist circumference. Baseline ESS was correlated with low HDL-cholesterol levels in our sample. Regarding anthropometric markers of insulin resistance, previous studies confirmed a relationship between EDS and greater waist circumference [25, 26]. The present study showed that liraglutide effectively reduced waist circumference, finally accompanied by a significant decrease in the mean ESS score.

In this context, an important perspective to consider is whether sleep/wake patterns could be partly modulated by food intake, especially with a high-fat dietary intake. Findings from several studies conducted in animal models demonstrated that high-fat diet and weight gain were associated with impaired sleep patterns, with more sleep time during the animals’ subjective day [27–29]; Wells et al. explored in a small group of adult volunteers (*n* = 16) the effects of a meal on objective and subjective measures of daytime sleepiness, showing that regular ingestion of fat-rich meals and significant excess of nutrients can predict EDS and poor quality of nocturnal sleep [30]. Clinical data showed reduced food intake, enhanced satiety and subsequent reductions in body weight were achieved upon the administration of GLP-1ra in patients with type 2 diabetes [31] and non-diabetic adults [32]. Interestingly, in a small study conducted with 20 obese and type-2 diabetic Japanese subjects, short-term treatment with liraglutide effectively reduced visceral fat adiposity, appetite and the urge for fat intake [33]. Collectively, we could contemplate that GLP-1ra liraglutide could improve EDS not only as a result of weight reduction, but also by decreasing appetite and reducing fat intake.

Our study participants were obese and had type-2 diabetes. After three months of treatment with liraglutide 75 % of subjects resulted in the reduction of both HbA1c and body weight, 17.5 % of patients revealed weight reduction but deterioration of HbA1c, while 2.6 % of subjects presented improvement in HbA1c levels but weight gain (Fig. 2a). These results entail that GLP-1ra liraglutide exerts its glucose-lowering action and improvement of body weight through different mechanisms. Regulatory agencies and international guidelines, such as the National Institute for Health and Care Excellence (NICE guidelines) [34] for the management of type 2 diabetes recommend to promote not only HbA1c reduction, as an indicator of metabolic improvement, but also weight loss (even at a lesser degree) as definition of “responders” to a GLP-1ra treatment for type 2 diabetes. Therefore, long-acting GLP-1ra liraglutide therapy appears to be a promising new agent for the management of ASPs in obese subjects with type 2 diabetes, especially when weight loss is a major concern.

Some limitations of this study need to be highlighted when interpreting the results. It should be noted that data from sleep studies (i.e. polysomnography) are lacking. The reason behind is that the study was aimed to reflect merely routine clinical practice, and no interventions or new procedures other than physicians’ daily routine were conducted. However, in light of all the promising results obtained, further prospective clinical studies should be conducted addressing this clinical endpoint. It is also worth acknowledging that the present study is an observational study, but not a randomized clinical trial. Although conducting observational studies are of great relevance to learn about the conditions derived from the routine clinical practice setting, they do not provide strong evidence. Furthermore, other factors in a free living environment might impact on the results, such as diet or exercise. Finally, it can be ascertained from the lack of a comparator group and the retrospective nature of the study design that biases could have been introduced when collecting data.

## Conclusions

In conclusion, three months of treatment with liraglutide effectively reduce body weight and waist circumference and ameliorates the glycaemic control measurements and lipid parameters. Concurrently, an improvement in mean ESS score was also achieved. Nevertheless, further research is needed to confirm and expand these preliminary results.

## Abbreviations

ASP: Abnormal sleep patterns
BMI: Body mass index
EDS: Excessive daytime sleepiness
ESS: Epworth sleepiness scale
FPG: Fasting plasma glucose
GLP-1: Glucagon-like peptide-1
HbA1c: Glycated haemoglobin
HDL: High-density-lipoprotein cholesterol
LDL: Low-density-lipoprotein cholesterol
MBG: Mean blood glucose
OSA: Obstructive sleep apnea
SD: Standard deviation
TC: Total cholesterol
TG: Triglycerides
